# Positive effects of laparoscopic sacrocolpopexy on anterior lateral defects and stress urinary incontinence

**DOI:** 10.1007/s00404-025-08214-0

**Published:** 2025-10-17

**Authors:** Norbert Nosal, Sebastian Ludwig, Markus Huebner

**Affiliations:** 1 Gynecology and Obstetrics, Elisabeth Hospital Essen, Klara-Kopp-Weg 1, 45138 Essen, Germany; 2 Faculty of Medicine and University Hospital of Cologne, Department of Gynecology and Oncology, Division of Urogynecology, University of Cologne, Cologne, Germany; 3 Department of Obstetrics and Gynecology, Medical Center-University of Freiburg, Faculty of Medicine, University of Freiburg, Freiburg, Germany

**Keywords:** Sacrocolpopexy, Cystocele, Central defect, Lateral defect, Stress urinary incontinence

## Abstract

**Objective:**

Laparoscopic sacrocolpopexy (SCP) is considered the gold standard for the correction of middle compartment (apical) prolapse. The extent to which simultaneous correction of the anterior compartment is necessary remains unclear. The aim of this study was to examine the anatomical and functional outcomes of the SCP on the anterior compartment with respect to the correction of central and lateral defects as well as stress urinary incontinence (SUI).

**Methods:**

This was a retrospective clinical follow-up with records of recurrences and complications as well as effects on SUI after laparoscopic SCP with and without simultaneous vaginal correction at Elisabeth Hospital, Essen, Germany, from 2014–2020 in 65 of 86 patients, with an average follow-up of 3.3 years.

**Results:**

Without simultaneous vaginal correction of the anterior compartment, recurrence was more common in women with central defect POP-Q Ba > − 2.0 [4 of 8 patients without correction (50% recurrence) vs. 1 of 23 patients with correction (4% recurrence)]. The OR for recurrence with surgery was 0.05 (95% CI 0.01; 0.52, *p* < 0,001). In women with lateral defects of the anterior compartment with a POP-Q Ba > − 2.0, simultaneous vaginal correction was not necessary, with a low recurrence rate (1 of 18 recurrences, 6%). Preexisting SUI grade ≥ 1 could be corrected in 27 of 36 patients. (75%, *p* < 0,001) according to the SCP.

Mesh erosion with reoperation (Clavien–Dindo IIIb) occurred in three patients (4.6%).

**Conclusion:**

Overall, this study suggests that patients with a central defect of the anterior vaginal wall can benefit from additional vaginal correction as part of the SCP. Furthermore, existing incontinence improves with the correction of prolapse.

## What does this study add to the clinical work

**Table:** 

Adding vaginal colporrhaphy to the SCP seems to be a viable method in women with anterior vaginal wall prolapse with a central defect. Although SCP is not considered to be SUI treatment, symptoms may improve by restoring the anatomy.	

## Introduction

**Fig. 1:**
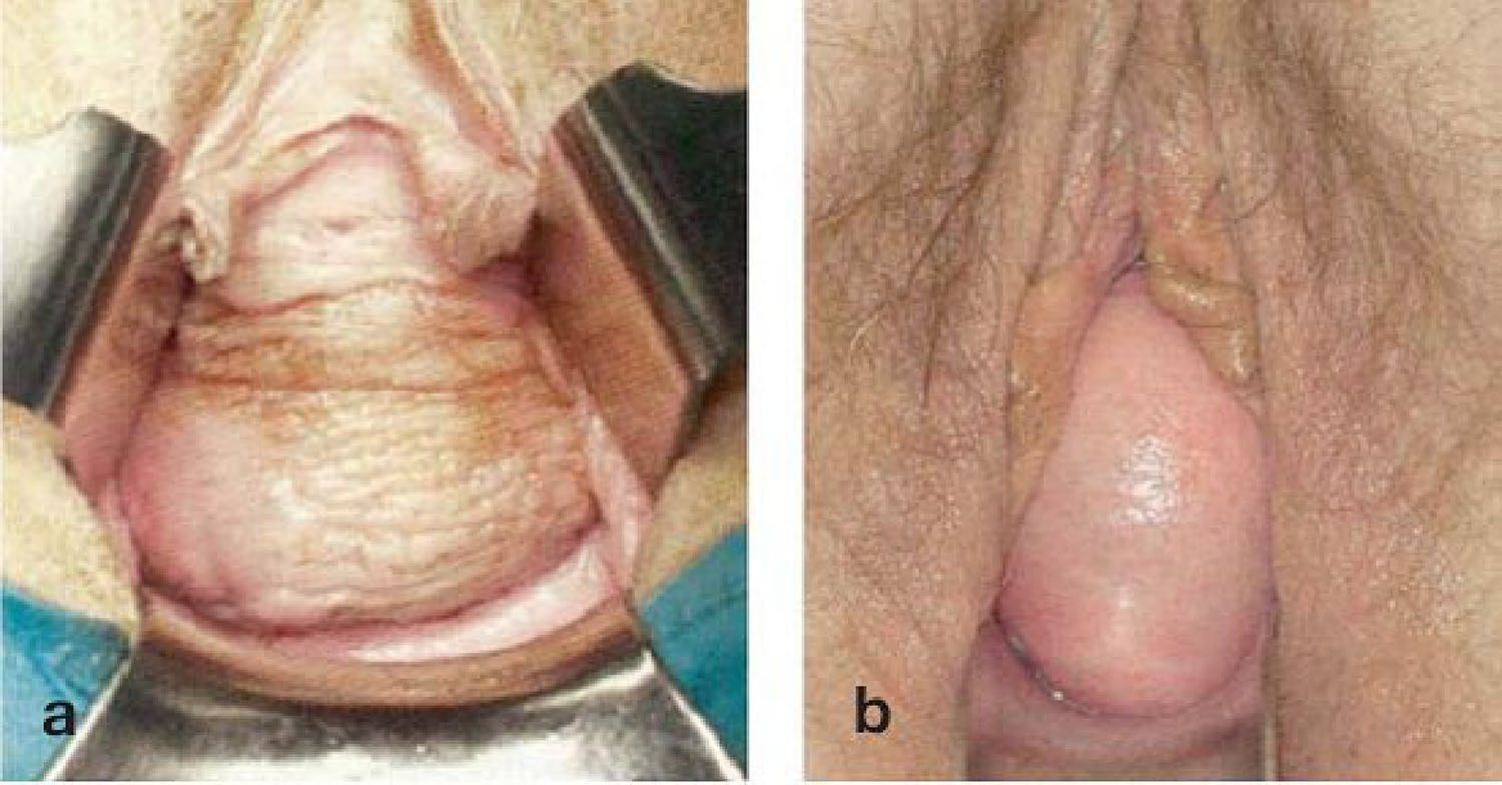
Pictures of lateral vaginal defects and central vaginal defects. Left image (**a**) showing typical rugae vaginae at the lateral vaginal defect. Right image (**b**) showing smooth vaginal epithelia at the central vaginal defect. Pictures from [6]

Instability of the pelvic floor can lead to prolapse of the pelvic organs into the vagina. The prevalence is between 2.9% [1] and 8.7% [2] of the total adult female population. It is very likely that the prevalence is significantly higher in up to 2/3 of all women [3] and increases with age, parity and weight [1, 2]. This can lead to prolapse of either the anterior vaginal wall (bladder, urethra) and/or the middle compartment (cervix and uterus, vaginal vault) and/or the posterior wall (rectum, bowel) [4]. Furthermore, a distinction can be made in the anterior compartment between a central and lateral defect [5, 6] (Fig. 1). Surgical interventions for prolapse have been described for more than one hundred years [7]. The surgical repair of suspension defects of the middle compartment (Level I according to DeLancey [8]) is particularly important, as it often complements corrections in the anterior or posterior compartment and can also be used as the sole operation for uterine or vaginal vault prolapse [9]. There are various vaginal operations, such as fixation of the uterosacral ligaments (USLs) or sacrospinous ligaments (SSLs), and abdominal/laparoscopic operations, such as SCP with and without uterine preservation [9]. In a systematic review, 21 studies evaluating laparoscopic SCP were analyzed, resulting in a cumulative success rate of 91% [10]. SCP is an effective procedure for treating vault prolapse. Polypropylene mesh is the preferred graft. Vaginal procedures for vault prolapse are well described and are suitable alternatives for those not suitable for SCP [10].

If laparoscopic fixation of the highest point of the vaginal apex (Level I) is performed, this is referred to as, depending on the condition, a sacrocolpopexy, a sacrocervicopexy or a sacrohysteropexy [11–14]. The operation can be performed in combination with vaginal correction of the anterior and posterior compartments so-called native tissue repair [9].

Apical location, paravaginal location, and hiatus size are highly correlated and are strong predictors of cystocele presence and size [15].

To date, few studies have explicitly described the surgical correction of cystoceles between central vaginal fascia defects (pulsation cystoceles, central descent, central smooth vaginal skin) and lateral defects of the suspensory ligaments (traction cystoceles, lateral descent, preserved rugae) [5, 6, 9] (Fig. 1).

Studies investigating the influence of apex stabilization on continence with and without simultaneous anterior vaginal correction are not available. The aim of this study was to investigate the influence of laparoscopic SCP on the possible recurrence rates of the three compartments. In addition to the apex and posterior compartments, a distinction was made, particularly in the anterior compartment, between the central and lateral defects. Furthermore, the effects of prolapse correction on existing urinary incontinence and possible complications were investigated.

## Methods

Between September 2014 and December 2020, 86 operations on women with genital prolapse were performed by one surgeon at the tertiary center of Elisabeth Hospital in Essen, Germany. After taking a medical history, a standardized ICIQ-UI SF questionnaire, a clinical examination with POP-Q assessment, vaginal palpation and pelvic floor sonography were performed. The severity of the prolapse was classified according to the POP-Q system [16]. All women were initially offered conservative treatment via vaginal estriol, pelvic floor training and pessary therapy. Women who opted for surgical treatment were informed about various surgical options, such as vaginal and laparoscopic surgery (SCP). If a cystocele or rectocele was present at the same time, the patient was informed about vaginal repair with absorbable sutures in terms of the anterior and posterior colporrhaphy. A possible postoperative worsening of SUI (so-called “pinch” [17]) or de novo urinary incontinence was noted with an offer of a repeat presentation for sequential treatment of the incontinence.

If the patient opted for the mesh-supported laparoscopic procedure, DynaMesh®-PR soft 4 × 18 cm was used. The medical device has a CE certificate and was used within the scope of the intended purpose and regular treatment practice. The product has been on the market since July 2012. The PVDF mesh was sutured laparoscopically depending on the tissue of the apex with 4 polypropylene sutures (braided, nonabsorbable). The mesh was placed completely retroperitoneally to replace the right uterosacral ligament, medial to the right ureter and lateral to the sigma and rectum. The required length was determined intraoperatively by applying maximum tension and releasing the tension by approximately 1 cm. The mesh was fixed at the cranial end by using 4 to 5 titanium screws/helices (ProTack Device, Covidien/Medtronic) used for fixation at the longitudinal ligament of the sacrum [18].

From September 2021 to February 2022, retrospective file-based data collection was carried out, and a study-specific questionnaire was issued with a follow-up examination of all patients. Consultation and approval were obtained from the Ethics Committee of North Rhine Medical Association (ÄEKNO, Germany) in accordance with §15 of the Professional Code of Conduct for Physicians Approval (No. 2021039).

Pelvic organ prolapse was quantified via the POP-Q system as described originally by Bump et al. [16]. In addition, central and lateral defects were classified as smooth vaginal epithelia, and lateral vaginal defects were classified as typical rugae vaginae (Fig. 1) [19, 20]. The SUI was recorded via a standardized ICIQ-UI SF questionnaire [21]. The Clavien‒Dindo classification was used to record complications [22]. The odds ratio (OR) was calculated via Fisher’s exact test for simultaneous vaginal anterior correction at the central vaginal defect. The data were analyzed via SAS 9.4. In addition, we used an in-house questionnaire to subjectively answer (yes or no) the following questions (Table 3): satisfaction with the operation result, choice of this operation again, recommendation, urinary problems before the operation, improvement of urinary problems after the operation, less nocturia after the operation and sexual activity.

## Results

Sixty-five of 86 patients (76%) participated in the follow-up examination. Eleven patients could not be reached, and another 10 declined to participate for personal reasons but stated by telephone that there was no prolapse recurrence or complications. The average time between surgery and follow-up was 3.3 years, with an average age of 52 years at the time of surgery (further data =  > Table 1).

**Table 1:** Characteristics of 65 patients treated for prolapse at Elisabeth Hospital, Essen, Germany, from 2014–2020

Values (median, Q1, Q3)	Overall cohort (*n* = 65)	Subgroups Vaginopexy (*n* = 6)	Cervicopexy (*n* = 27)	Hysteropexy (*n* = 32)
Age *years*	52.0 (48.0;56.0)	61.5 (52.0;69.0)	50.1 (48.0;52.0)	51.9 (44.0;58.0)
Height *cm*	166 (162;170)	164 (159;169)	167 (163;172)	165 (160;169)
Weight *kg*	73.3 (66.0;79.0)	71.5 (68.0;76.0)	71.3 (62.0;75.0)	75.4 (68.0;81.0)
BMI	26.4 (23.0;29.0)	26.8 (26.0;29.0)	25.4 (22.0;27.0)	27.3 (24.0;30.5)
ASA	2.0 (2.0;2.0)	2.0 (2.0;2.0)	2.1 (2.0;2.0)	1.8 (1.5;2.0)
Vaginal delivery (abs.)	2.2 (2.0;3.0)	2.7 (2.0;4.0)	2.2 (2.0;3.0)	2.2 (1.0;3.0)
Sectio	0.2 (0;0)	0	0.2 (0;0)	0.2 (0:0)
Years after surgery	3.3 (2.0;4.0)	2.8 (2.0;4.0)	3.7 (2.0;4.0)	3.2 (2.0;4.0)

The characteristics of the cohort (*n* = 65) described the mean and Q1/Q3 values for age (in years), height (in cm), height (in kg), BMI (body mass index), ASA (American Society of Anesthesiologists physical status classification system), vaginal delivery and sectio, and years between surgery and examination

In 6 patients, the uterus had already been removed before the laparoscopic SCP. In 27 patients, a sacrocervicopexy was performed, in which, in 6 of the 27 patients, the supracervical hysterectomy had already been performed before the prolapse surgery. A further 32 patients underwent uterus-preserving sacrohysteropexy (Table 1).

A total of 49 of 65 patients (75%) had concomitant anterior compartment prolapse, with a POP-Q Ba mean of 0.2 (SD 0.4) (Table 2).

**Table 2:** Values before and 3.3 years after surgery among 65 women with prolapse, Elisabeth Hospital Essen, Germany, 2014–2020

Value (mean, 2σ deviation)	*n*	POP-Q before	POP-Q after	POP-Q Diff
Apex defect (POP-Q C)	65	− 1.8 (0.7)	− 7.8 (0.3)	− 6.0 (0.7)
Anterior defect (cystocele) (POP-Q Ba)	49	0.2 (0.4)	− 2.2 (0.3)	− 2.4 (0.5)
Central defect of cystocele	31	0.4 (0.6)	− 1.9 (0.5)	− 2.3 (1.0)
Lateral defect of cystocele	18	− 0.8 (0.5)	− 2.8 (0.2)	− 2.0 (0.7)
Posterior defect (rectocele) (POP-Q Bp)	17	− 0.4 (0.4)	− 2.3 (0.6)	− 1.9 (1.1)
SUI (0–3°)	33	1.5 (0.2)	0.2 (0.2)	− 1.3 (0.2)

The values indicate the *2σ* deviation of prolapse depending on the compartment and incontinence. Three compartments, the apex, anterior defect and posterior defect, were examined before and after surgery for a mean of 3.3 years. The anterior defect was separated into central and lateral defects. All three compartments were separated by POP-Q: apex score − 8 to + 8 and anterior and posterior scores − 3 to + 3. On the incontinence scale 0–3, all scores ≥ 1 were recorded before and after surgery

Among the 49 patients with anterior prolapse, 31 patients had central anterior vaginal defects, with a mean POP-Q Ba of 0.4 (SD 0.6) and a mean postoperative POP-Q Ba of -1.9 (SD 0.5). Among these patients, 23 patients were treated with vaginal anterior correction, and 22 patients experienced no recurrence at follow-up (96%). Among the eight patients who, despite a central defect, did not receive a vaginal anterior correction—at their own request because of refusal of vaginal surgery—four patients showed a postoperative correction of the central defect due to the SCP of POP-Q Ba ≤ − 2.0. However, four patients had persistent central defects with POP-Q Ba > − 2.0. The odds ratio obtained via Fisher’s exact test of postoperative recurrence after surgery for anterior vaginal defects was 0.05 (95% CI 0.01; 0.52).

**Fig. 2:**
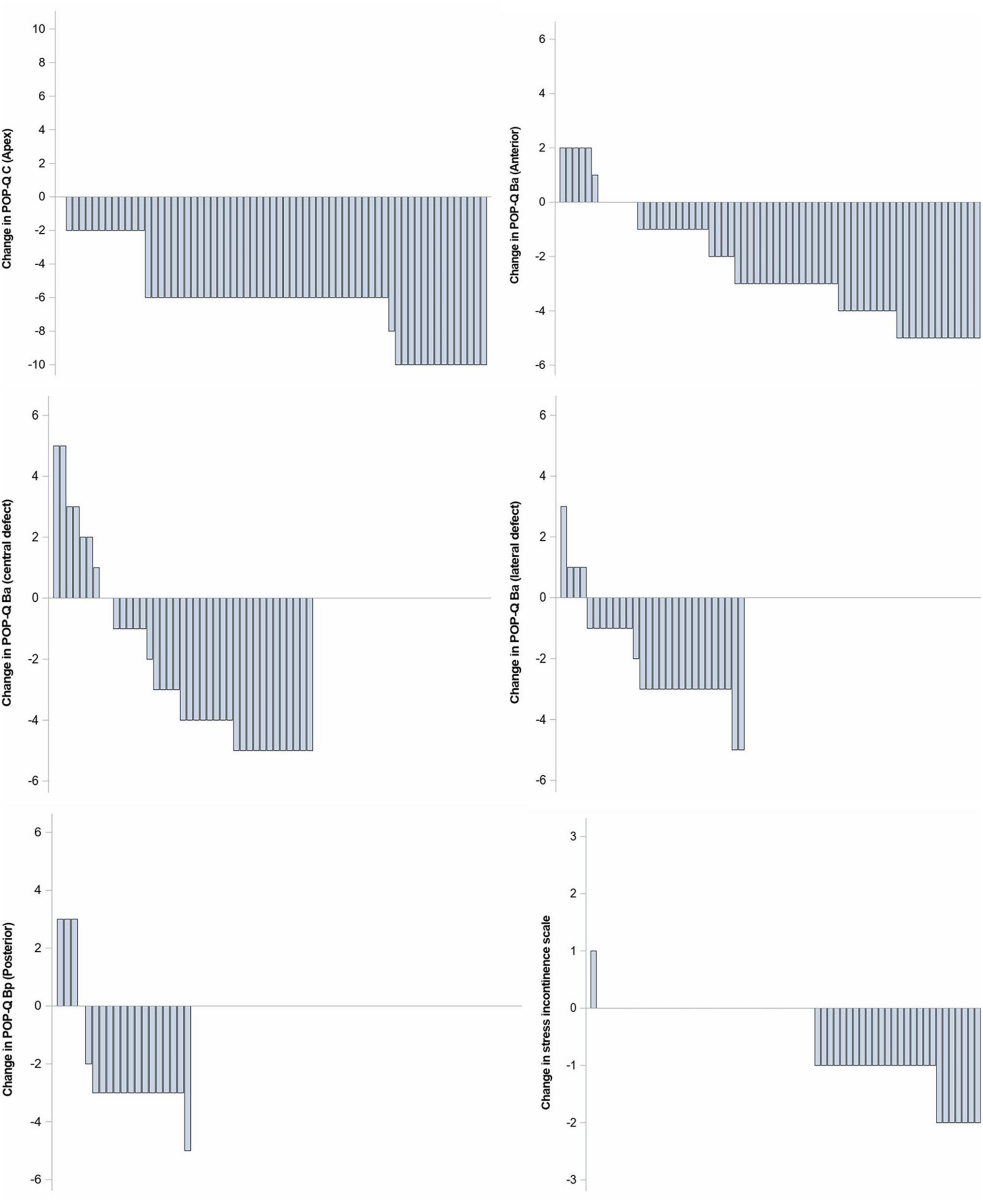
Changes in POP-Q scores before and after surgery in 65 patients at Elisabeth Hospital Essen, Germany, 2014–2020. Changes in POP-Q points (y-axis) in the waterfall diagram for 65 patients (x-axis). Each column represents a patient change before and after surgery. The upper left panel shows C (apex − 8 to 8), upper right Ba (anterior − 3 to 3), middle left Ba (central defect − 3 to 3), middle right Ba (LATERAL defect − 3 to 3), lower left Bp (posterior − 3 to 3) and lower right SUI level − 3 to 3

The remaining 18 of 49 patients with anterior prolapse had a lateral anterior vaginal defect with a POP-Q Ba mean of − 0.8 (SD 0.6). In these groups, no vaginal correction was performed, only a laparoscopic SCP to reinforce Level I (POP-Q C) was used. The postoperative POP-Q score was Ba -2.75 (SD 0.25) (Fig. 2, middle right).

Urinary incontinence (grades 1–3) was recorded preoperatively. A total of 36 patients (55%) reported urinary incontinence, 33 (51%) reported mild and moderate SUI [1.5 on a scale of 0–3 (SD 0.2)], and 3 patients reported urgency urinary incontinence. Among the 33 patients with preoperative SUI, only 7 had persistent postoperative incontinence, and no patients had postoperative urgency urinary incontinence (Fig. 2 lower right) (further data =  > Tab 2).

The following possible complications were denied: urinary tract infections, urinary retention, constipation, fecal incontinence, fistula, permanent or recurrent pain, and de novo dyspareunia. Three cases of vaginal mesh erosion requiring reoperation (Clavien–Dindo IIIb) were recorded, one after SCP within one year and two after sacrocervicopexy two years after surgery. In all the patients, the mesh was partially removed laparoscopically. There were no mesh complications after sacrohysteropexy.

A questionnaire was distributed, and the results are described in Table 3.

**Table 3:** Subjective characteristics of 65 patients after treatment for prolapse, Elisabeth Hospital Essen, Germany, 2014–2020

	Results (in n abs.)	Results (in %)
Subjective satisfaction with operation result	60	92
Choose this operation again	60	92
Recommendation	62	95
Urinary problems before operation	40 of 65	62
Improvement of urinary problems after operation	30 of 40	75
Less nycturia after operation	10 of 40	25
Sexual activity	43	66
Positive impact of operation on sexual activity	25 of 43	58

Postoperative questionnaire results for 65 patients who underwent surgery (absolute and relative percentages)

## Discussion

Laparoscopic SCP is a safe procedure with few complications for preventing prolapse recurrence. If the surgeons differentiate between a central and lateral defect in the case of a vaginal anterior defect, it seems that reinforcement of Level I by laparoscopic SCP appears to be sufficient to treat both apical and lateral defects, whereas in the case of a central defect, the SCP alone seems to be insufficient. One of the few long-term analyses investigating the recurrence of cystocele over at least five years (5.5–18 years follow-up period) revealed that 35 of 135 women (26%) experienced prolapse symptoms that recurred after previous surgical correction of the anterior compartment [23]. No distinction was made as to whether fixation of the apex was performed or whether the anterior defect was central or lateral, as described in Fig. 1. This means that there is also a lack of information on the recurrence rate of central and lateral defects. The lateral defect describes an avulsion of the arcus tendinous fasciae pelvis, whereas the central vaginal defect describes a weakness of the pubovaginal fascia [24]. An anterior vaginal correction should be performed in the case of a central defect, whereas a lateral defect requires a so-called lateral repair [24].

Anterior mesh fixation does not differentiate between central and lateral defects, as apical and lateral fixation points correct both vaginal defects. Notably, compared with laparoscopic mesh insertion, vaginal mesh insertion may cause more frequent mesh erosion and is therefore controversial among urogynecologists, leading to vaginal mesh bans in many countries [25].

Apical location, paravaginal location, and hiatus size are highly correlated and are strong predictors of cystocele presence and size [15].

**Fig. 3:**
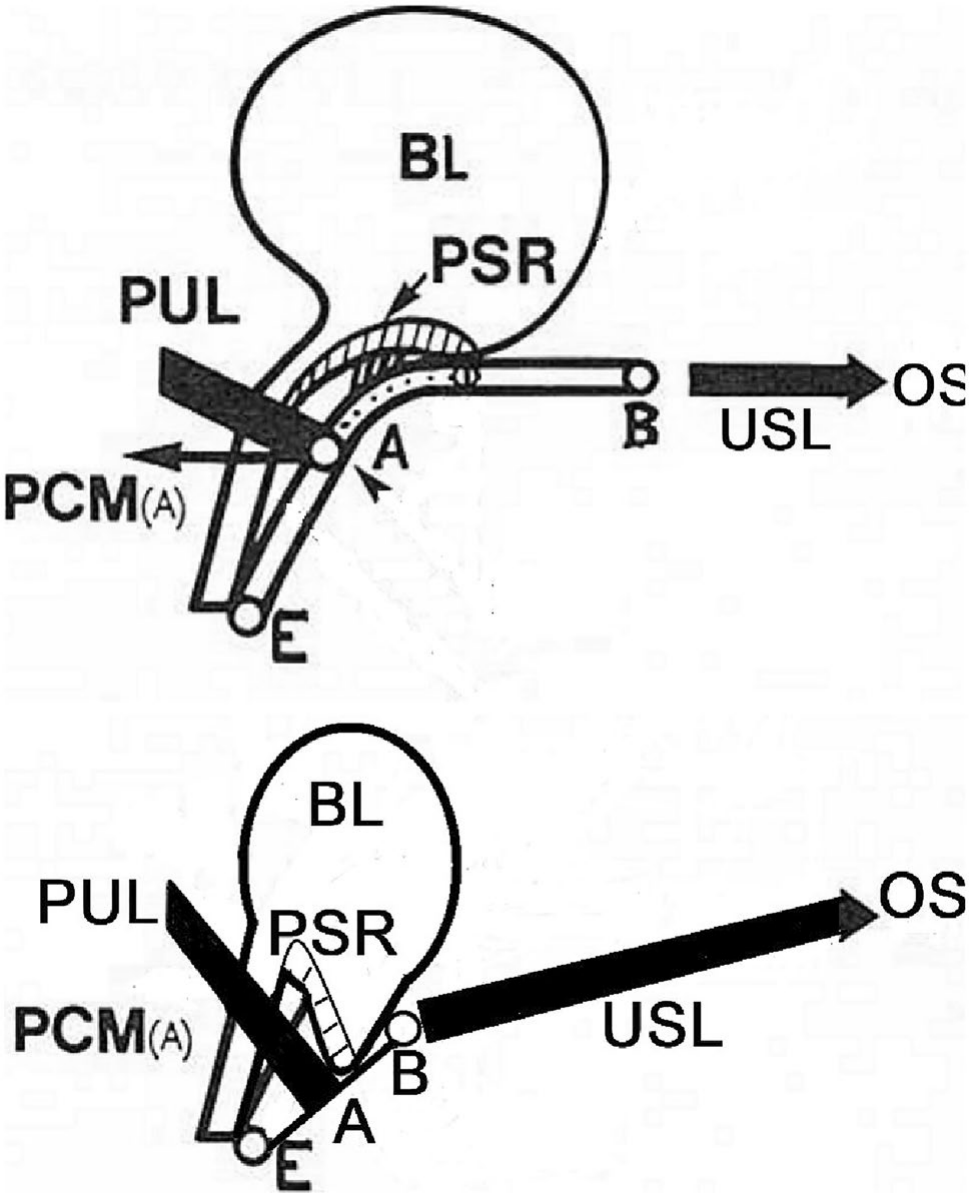
Scheme of the functional anatomy of the lower urinary tract with and without an intact uterosacral ligament. Upper picture: normal uterosacral ligament (USL) between the apex (**B**) and OS (os sacrum). Bladder (BL) and presumed stretch receptor (PSR) in the normal position, pubo urethral ligament (PUL) and anterior pubococcygeal muscle (PCM(A)) in short form with insertion in the vaginal wall (**A**). **E** Shows vaginal entry. Lower picture: loose USL and PUL with disabled PCM (**A**) and anterior vaginal wall defect. Both images were modified from Petros and Ulmsten, 1990 [29]

Chen et al. described a collinear triad consisting of apical location, paravaginal location, and hiatus size, which were not only the strongest predictors of cystocele size, but also highly correlated with one another [15]. On the basis of these data, the isolated elevation of the apex by laparoscopic SCP in our population corrected 96% of the lateral vaginal defects but only 50% of the central vaginal defects. This is most likely due to vaginal stretching (point B in Fig. 3) by replacing the utero-sacro-ligament (USL) and consequently also tightening the pubourethral ligaments (PUL) and probably also the lateral vaginal structures of the arcus tendinous fasciae pelvis. In contrast, reinforcement at Level I is not sufficient in the case of a central vaginal defect, as the tensile force of the SCP in the area of the pubovaginal fascia does not appear to be sufficient. This leads to the conclusion that in the case of a central defect, vaginal correction should be performed at the simultaneous SCP to avoid recurrence of the anterior compartment. Similar results were also summarized in a short review by Page et al. [26]. Here, the vaginal apex was bilaterally stabilized along the left and right uterosacral ligaments via PVDF sutures. In addition to almost 100% apical reconstruction (POP-Q stage 0), urinary continence was restored in more than 40% of cases solely through apical reconstruction [18, 27, 28].

Incontinence is significantly improved by SCP with and without simultaneous vaginal anterior correction. An improvement in continence rates after surgical correction of genital prolapse has been described in other studies, with rates of up to 50% [9]. However, our data revealed a clear improvement in SUI of 74%. Petros and Ulmsten reported in 1990 (Fig. 3) that the main burden of the first closure mechanism is based on the stability of the PUL and the function of the anterior pubococcygeal muscles, which are influenced in terms of functionality by the flaccid vagina [29]. The second closure mechanism, according to Petros and Ulmsten, which bear the main burden of urinary incontinence, requires intact USL [29]. In our study, these USLs were replaced with mesh, and consequently, the vagina was tightened. Thus, the simultaneous stretching of the PUL and consequently the restoration of the functionality of the anterior pubococcygeal muscle (Fig. 3) is a possible mechanism of action for the elimination of urinary incontinence. Any urinary incontinence that exists before surgery for prolapse should therefore be treated in two stages because apical correction alone seems to improve urinary incontinence.

With respect to the recurrence rate of the middle compartment, our results correspond to the excellent results of laparoscopic SCP compared with those of other types of vaginal fixation. Other studies have confirmed the low complication rate of sacrohysteropexy compared with that of sacrocolpopexy and sacrocervicopexy [14, 30]. Noe, Bernard et al. 2021 argued that it is unclear why many surgeons continue to decide against uterine preservation and proceed with “reflex hysterectomy” in the case of prolapse [31–34].

A possible reason for uterine retention may be incomplete family planning in women of fertile age, whereby insufficient data should be pointed out in the case of incomplete family planning and SCP. A systematic review and metaanalysis showed no difference in the recurrence of POP following sacrohysteropexy using various apical suspension procedures [35]. Jefferis et al. described six pregnancies after sacrohysteropexy with uterine preservation [36]; in our cohort, there were three pregnancies without complications or vaginal deliveries after sacrohysteropexy.

The limitations of our study were the completely retrospective study design without blinding of the investigators.

The results show that SCP is a safe procedure with a low recurrence rate. Serious complications are rare overall, and in our population, none were observed if the uterus was preserved. In the context of a retrospective cohort study, the data provide an initial indication that an anterior vaginal central defect POP-Q Ba > − 2.0 should be surgically corrected in contrast to an anterior vaginal lateral defect POP-Q Ba > − 2.0. A further prospective, multicenter and randomized study is in progress.

## Data Availability

No datasets were generated or analyzed during the current study.
